# Integrated transcriptome and metabolome analysis reveals the molecular responses of *Pardosa pseudoannulata* to hypoxic environments

**DOI:** 10.1186/s40850-024-00206-y

**Published:** 2024-07-04

**Authors:** Jinjin Li, Yun-e Tang, Bo Lv, Juan Wang, Zhi Wang, Qisheng Song

**Affiliations:** 1 College of Life Science, Hunan Normal University, Changsha, Hunan 410006 China; 2 Division of Plant Sciences and Technology, University of Missouri, Columbia, MO 65211 USA

**Keywords:** *Pardosa pseudoannulata*, Hypoxic stress, Energy metabolism, Metabolome, Transcriptome

## Abstract

Terrestrial organisms are likely to face hypoxic stress during natural disasters such as floods or landslides, which can lead to inevitable hypoxic conditions for those commonly residing within soil. *Pardosa pseudoannulata* often inhabits soil crevices and has been extensively studied, yet research on its response to hypoxic stress remains unclear. Therefore, we investigated the adaptive strategies of *Pardosa pseudoannulata* under hypoxic stress using metabolomics and transcriptomics approaches. The results indicated that under hypoxic stress, metabolites related to energy and antioxidants such as ATP, D-glucose 6-phosphate, flavin adenine dinucleotide (FAD), and reduced L-glutathione were significantly differentially expressed. Pathways such as the citric acid (TCA) cycle and oxidative phosphorylation were significantly enriched. Transcriptome analysis and related assessments also revealed a significant enrichment of pathways associated with energy metabolism, suggesting that *Pardosa pseudoannulata* primarily copes with hypoxic environments by modulating energy metabolism and antioxidant-related substances.

**Supplementary Information:**

The online version contains supplementary material available at 10.1186/s40850-024-00206-y.

## Introduction

Oxygen (O_2_) is essential for life in the biosphere. Research on O_2_ in humans began in the 1770s [1]. However, interest in the effects of hypoxia on organisms emerged in the mid-19th century [2]. Oxygen deficiency can disrupt the physiological functions of aerobic organisms. For example, under hypoxic conditions, the blood clam (*Tegillarca granosa*) exhibits significant reductions in total hemocyte counts, hemoglobin concentrations, and intracellular reactive oxygen species (ROS) levels, leading to immunotoxicity [3]. Meanwhile, hypoxia can also disrupt the energy metabolism of *Crustacea* [4]. In recent years, there has been significant progress in studies on how arthropods cope with hypoxic environments. For instance, *Drosophila melanogaster* triggers an autophagic response under low oxygen conditions, and some terrestrial insects experience delayed growth and development [5, 6]. Additionally, in hypoxic conditions, *Macrobrachium nipponense* experiences changes in gut microbiota and mucosal morphology, with decreased activity of intestinal immune enzymes and damage to its energy metabolism and antioxidant systems [7–10].

Organisms have adapted and developed various survival strategies to cope with hypoxic conditions [11]. In arthropods, this often involves regulating energy metabolism to manage low oxygen stress [12–14]. Alternatively, activating key antioxidant enzyme activities can also help manage hypoxic stress [15]. These studies not only enrich our understanding of arthropod physiological ecology but also provide important perspectives for predicting the impact of environmental changes on biodiversity. However, despite extensive research covering various arthropods, the adaptive mechanisms of arachnids like spiders in hypoxic environments remain relatively unknown. This is particularly crucial for species like *Pardosa pseudoannulata*, which play key roles in agricultural ecosystems, where understanding their survival strategies in extreme environments is particularly important.

*P. pseudoannulata* is a dominant spider species of paddy fields and a significant predator of various agricultural pests such as rice planthoppers and leafhoppers, playing a vital role in pest control within rice fields [16–18]. *P. pseudoannulata* has a wide habitat range, living not only in wetlands, meadows, and rice fields but also at altitudes around 3500 m where oxygen concentrations are lower, and even in soil crevices [19, 20]. Although terrestrial environments generally offer easier access to oxygen than aquatic settings, terrestrial invertebrates or their tissues may still experience hypoxia across various habitats and environmental conditions [21, 22]. Especially during rainy seasons or floods, *P. pseudoannulata*’s typical habitats may face the risk of submersion, limiting oxygen supply and necessitating short-term survival in hypoxic conditions. While there is no shortage of studies on arthropods coping with hypoxic stress, the responses of spiders to hypoxic stress have not been sufficiently studied. Recent advances in next-generation high-throughput sequencing technologies have opened new avenues for studying responses and adaptation mechanisms at the molecular level [23, 24]. In this study, we used metabolomics and transcriptomics to investigate the adaptive strategies and physiological responses of *P. pseudoannulata* under hypoxic conditions. This not only enhances our understanding of its ecological functions but also aids in developing more effective eco-agricultural management strategies to promote the health and sustainability of rice field ecosystems.

## Results

### Hypoxic stress alters the metabolism of *P. pseudoannulata*

**Fig. 1:**
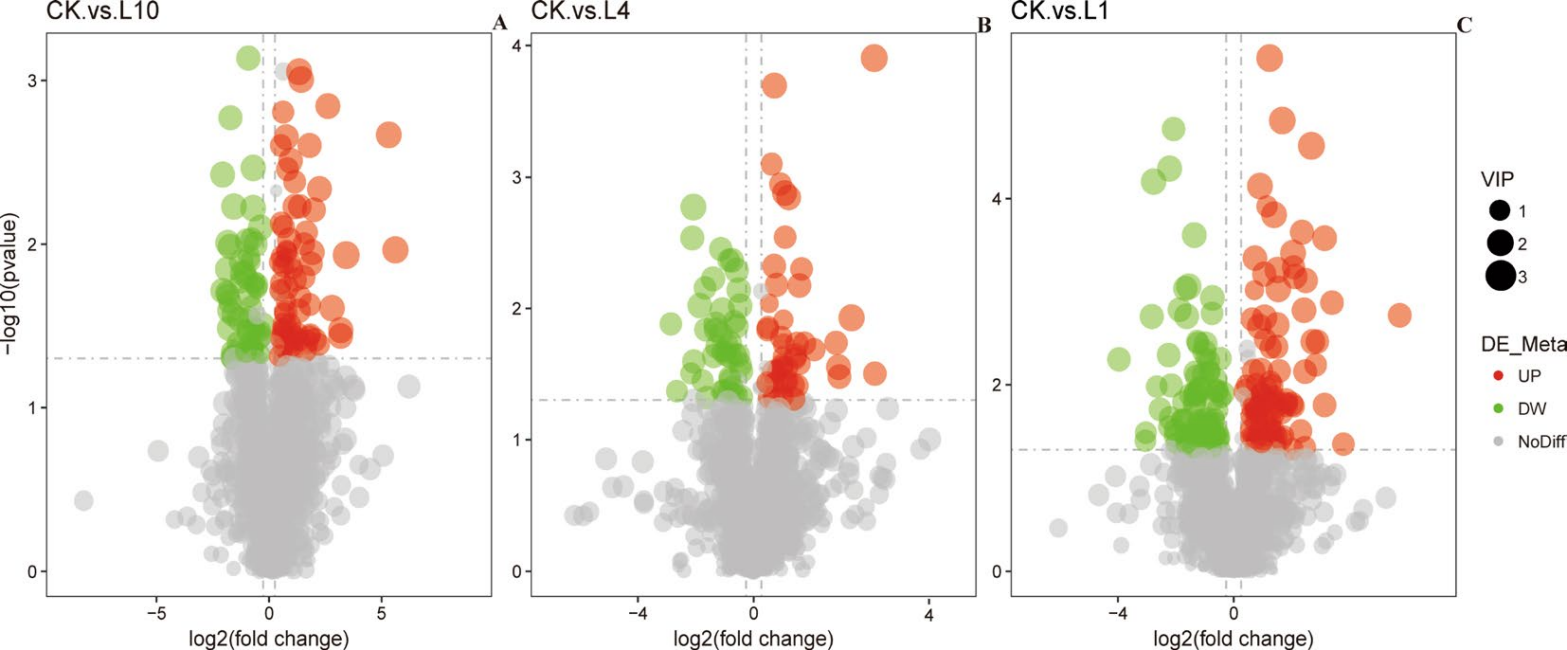
Differences in metabolite abundance under hypoxic conditions. The volcano plots illustrate variances between CK and the L10, L4, and L1 groups. Red dots indicate upregulation, green dots indicate downregulation, gray dots indicate no significant difference, and the size of the dots represents the VIP value

**Fig. 2:**
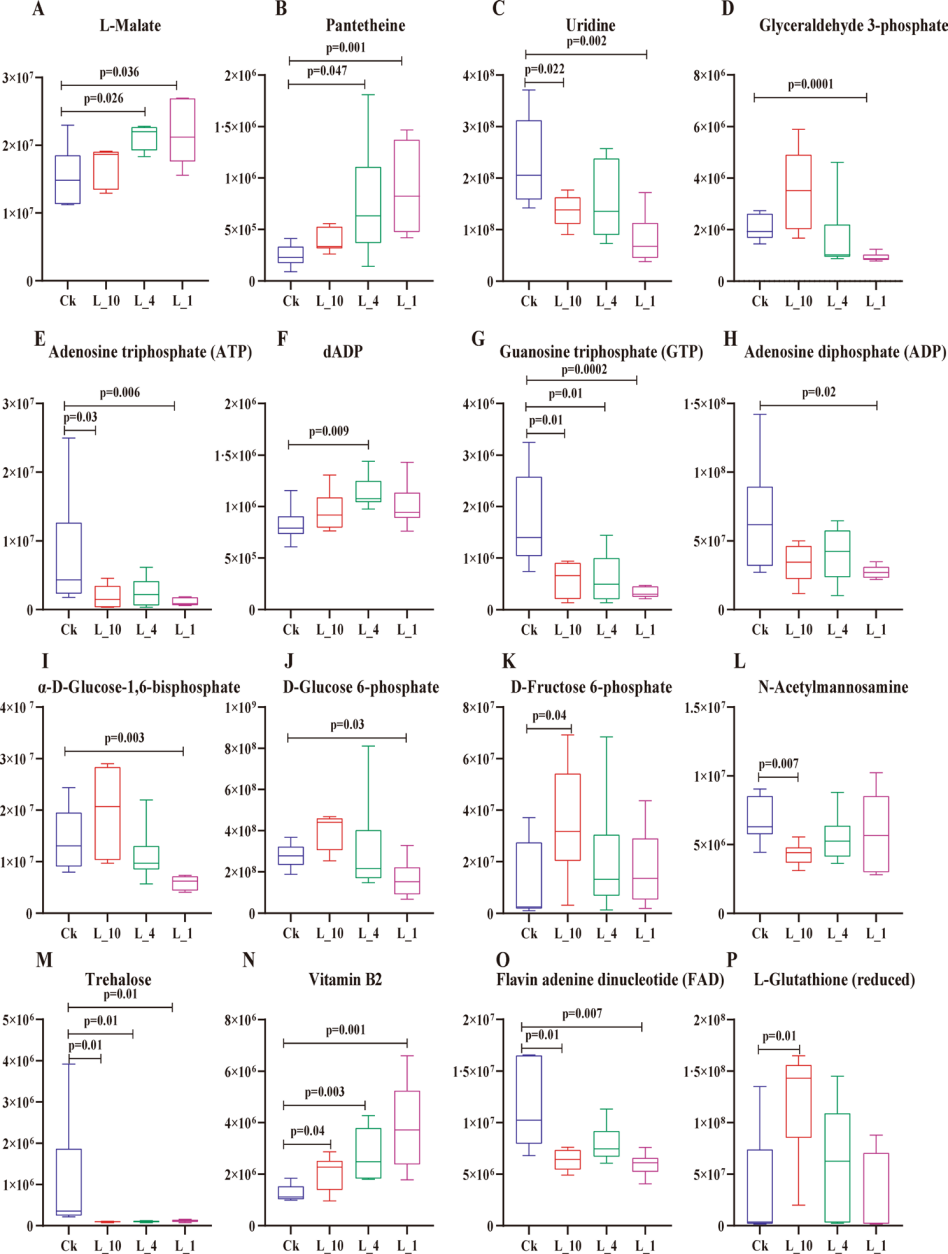
The impact of hypoxia on the metabolites of *P. pseudoannulata*. (**A**-**P**) Differential abundance of various DEMs. All values are presented as mean ± SEM

**Fig. 3:**
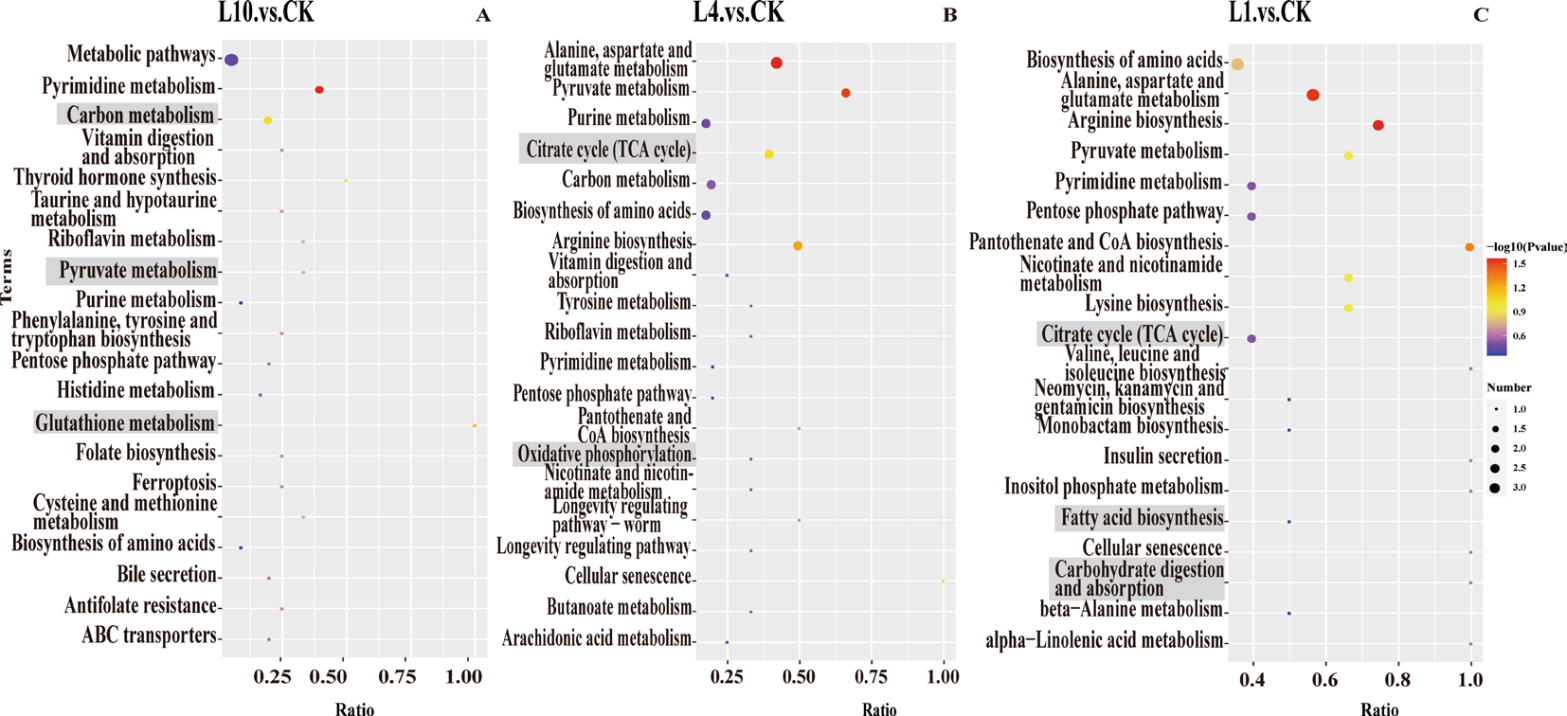
*P. pseudoannulata* metabolome KEGG enrichment analysis. (**A**-**C**) Top 20 KEGG enriched pathways for L10 vs. CK, L4 vs. CK, and L1 vs. CK. The Ratio is x/y (number of DEGs in the corresponding metabolic pathway/total number of identified metabolites in that pathway), where a higher value indicates a higher enrichment level of DEGs in that pathway. The color of the dots represents the p-value, with smaller values indicating greater reliability and statistical significance of the test. The size of the dots represents the number of DEGs in the corresponding pathway, with larger values indicating more DEGs in that pathway

We conducted comprehensive metabolomic analyses in both positive (pos) and negative (neg) ion modes on collected samples, observing significant metabolic profile changes in *P. pseudoannulata* under hypoxic stress. Metabolomic identification revealed a total of 1632 metabolites. Compared to the control group (CK), there were 68, 47, and 95 metabolites upregulated and 57, 46, and 94 metabolites downregulated at 10% (L10), 4% (L4), and 1% (L1) low oxygen concentrations, respectively (Fig. 1). In this set of metabolites, the abundance of L-Malate and Pantetheine significantly increased (Fig. 2A-B). Uridine and Glyceraldehyde 2-phosphate showed a significant decrease in abundance (Fig. 2C-D). Energy molecules such as ATP, GTP, and ADP exhibited a significant decrease in abundance, while dADP showed a significant increase (Fig. 2E-H). Certain sugars such as α-D-glucose-1, 6-bisphosphate, D-glucose 6-phosphate, N-acetylmannosamine, and trehalose demonstrated a significant decrease in abundance, whereas D-fructose 6-phosphate showed a significant increase (Fig. 2I-M). Some essential vitamins like vitamin B2 showed a significant increase in abundance (Fig. 2N). Coenzymes such as FAD displayed a significant decrease in abundance (Fig. 2O). In addition, to explore the potential impact of hypoxia on the metabolic pathways of DMEs, we conducted KEGG enrichment analysis on all differentially expressed genes(DEGs). Compared to the CK group, significantly enriched pathways in L10 included Carbon metabolism, Pyruvate metabolism, and Glutathione metabolism (Fig. 3A). In L4, significantly enriched pathways included the Citrate cycle (TCA cycle) and Oxidative phosphorylation (Fig. 3B). In L1, significantly enriched pathways comprised the Citrate cycle (TCA cycle), Fatty acid biosynthesis, and Carbohydrate digestion and absorption (Fig. 3C). This indicates that *P. pseudoannulata* can adapt to hypoxic stress by regulating the synthesis and utilization of its antioxidant substances and energy-related compounds.

### Hypoxia stress alters the gene expression in *P. pseudoannulata*

**Fig. 4:**
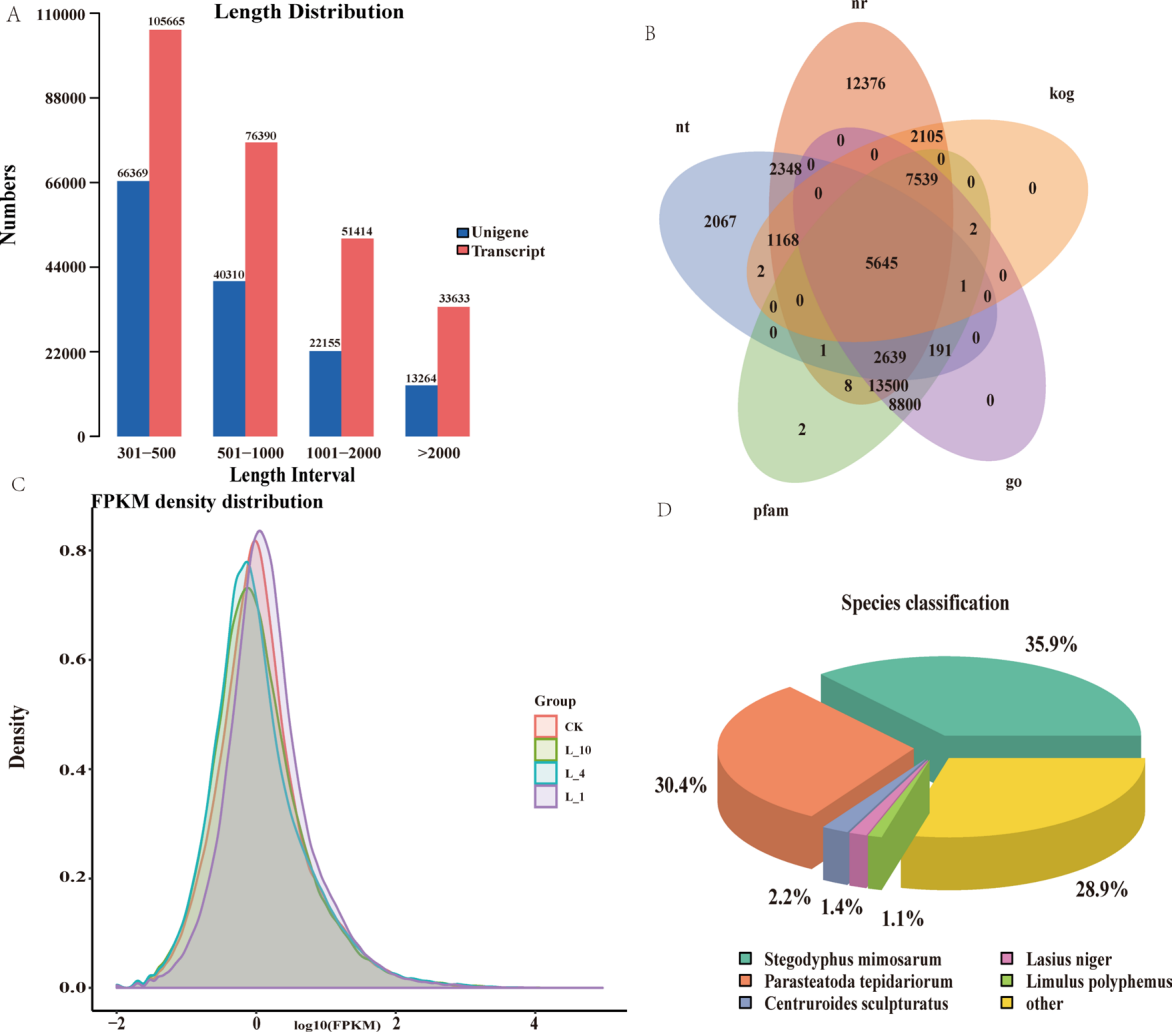
Map of transcriptome-related parameters. (**A**) Plot of transcript and unigenes length distribution. (**B**) Annotation status of unigenes in Nr, KOG, GO, Pfam, Nt databases. (**C**) FPKM density distribution map of unigenes. (**D**) Annotation status of unigenes in the nr database

Spiders from the L10, L4, L1, and CK groups were dissected under a stereo microscope to obtain their book lungs for transcriptome analysis. Each group consisted of three replicates, with each replicate containing 10 book lungs. A total of 267, 102 transcripts were obtained from transcriptome sequencing, ranging in length from 301 bp to 18, 984 bp, along with 142, 098 unigenes (Fig. 4A). The obtained unigenes were annotated in seven public databases, including Nr, Nt, KO, Swiss-Prot, Pfam, KOG, and GO, yielding 47, 329, 14, 062, 20, 579, 34, 409, 38, 328, 16, 462, and 38, 317 annotated entries, respectively (Fig. 4B). To assess the gene expression profiles among different treatment groups, gene expression levels were calculated based on FPKM values, revealing similar expression levels among the groups (Fig. 4C). Comparison with the Nr database indicated that the species distribution categories were mainly concentrated in spiders, such as *Stegodyphus mimosarum*, *Parasteatoda tepidariorum*, and *Nephila clavipe*, among others (Fig. 4D).

**Fig. 5:**
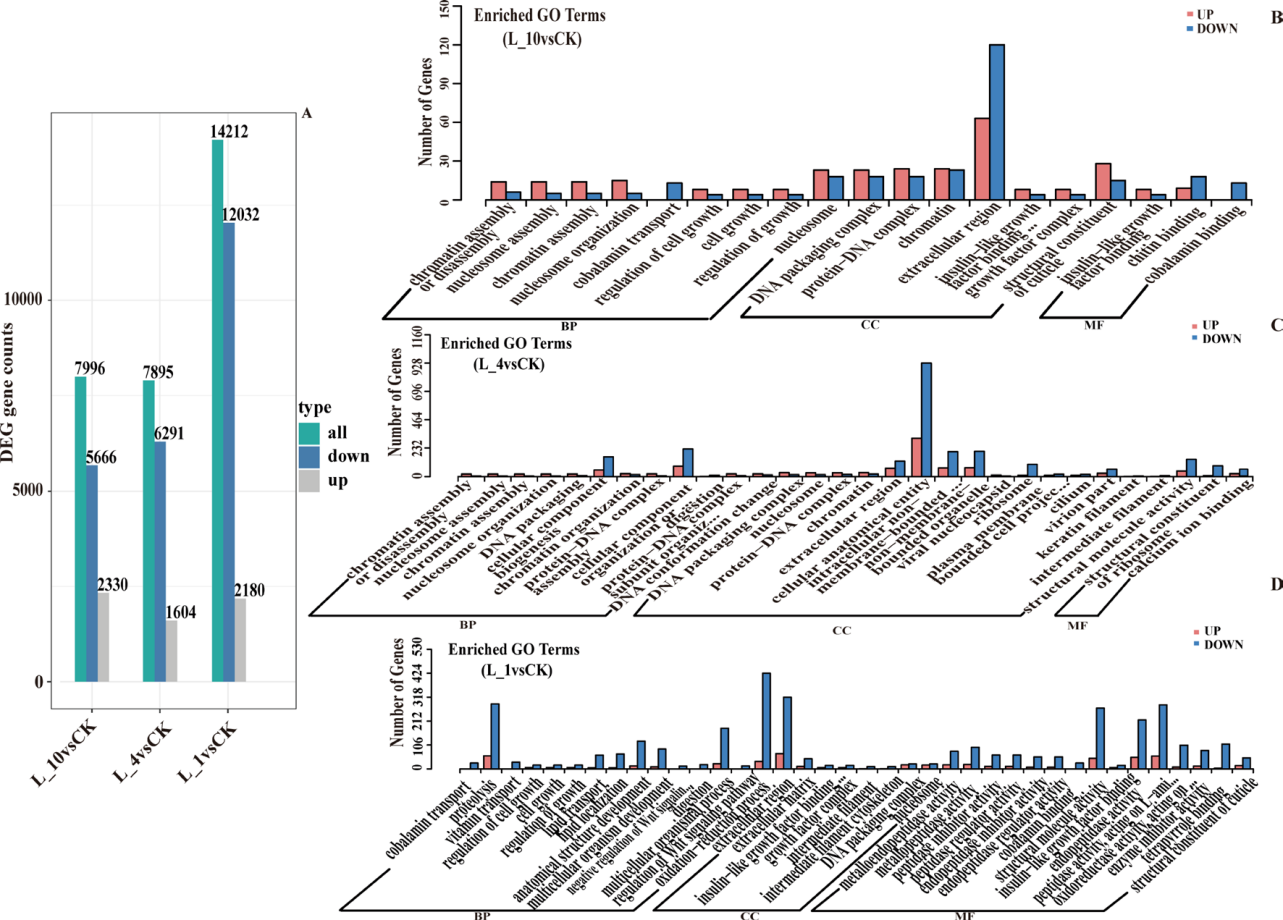
Histogram of DEGs in the book lung transcriptome of *P. pseudoannulata* and a secondary taxonomic map of GO enrichment of DEGs. (**A**) Bar graph of DEGs. (**B**-**D**) GO secondary categorization maps for DEGs in CK vs. L10, CK vs. L4, and CK vs. L1

**Fig. 6:**
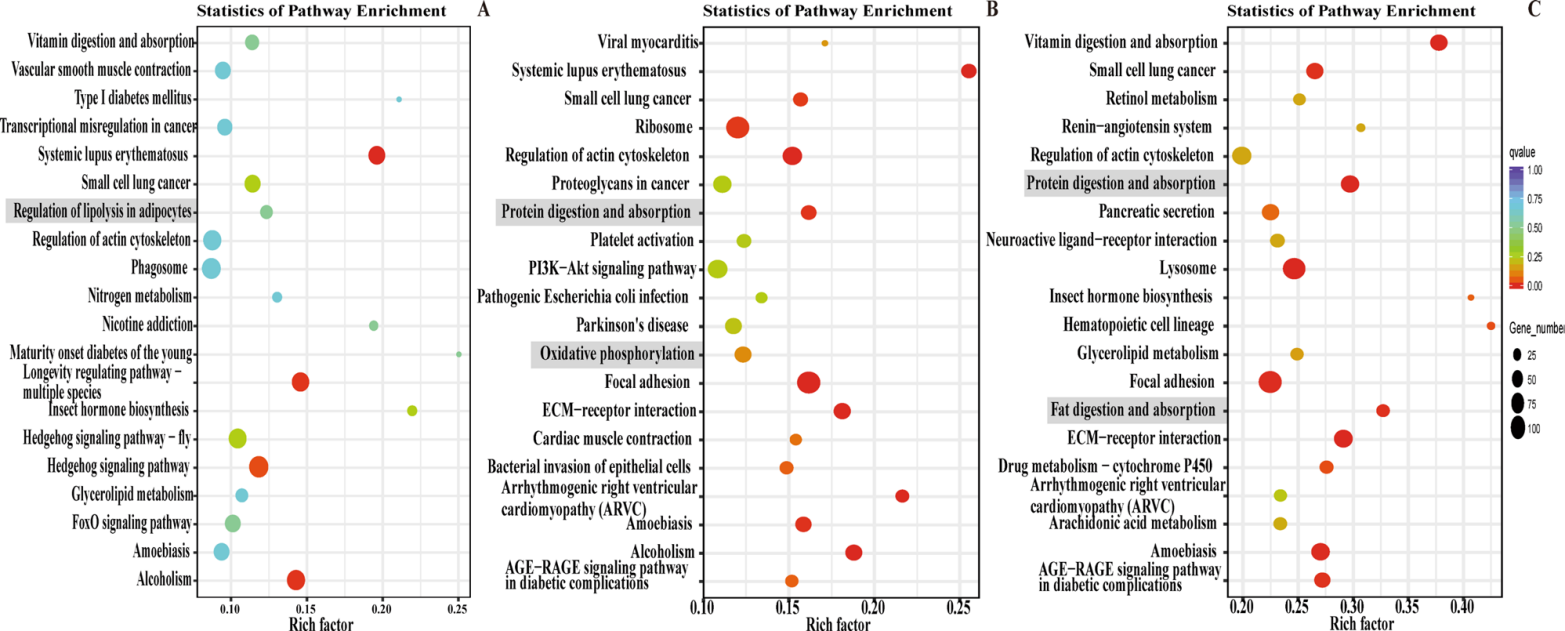
KEGG enrichment analysis of the book lung transcriptomes of *P. pseudoannulata*. (**A**-**C**) L10 vs. CK, L4 vs. CK, and L1 vs. CK were the top 20 KEGG-enriched pathways. The enrichment factor indicates the ratio of the number of DEGs to the number of all unigenes

In addition, according to the transcriptome data, there were 2330, 1604, and 2180 upregulated genes, as well as 5666, 6291, and 12,032 downregulated genes in L10, L4, and L1, respectively (Fig. 5A). To investigate the effect of hypoxia on the respiratory function of *P. pseudoannulata*, we conducted Gene Ontology (GO) and Kyoto Encyclopedia of Genes and Genomes (KEGG) enrichment analysis on the DEGs. The enrichment analysis results indicated that in L10, the main GO enrichment terms included extracellular region (cellular component), chromatin (cellular component), and structural constituent of cuticle (molecular function) (Fig. 5B). In L4, the main GO enrichment terms included cellular anatomical entity (cellular component), cellular component organization (biological process), and cellular component biogenesis (biological process) (Fig. 5C). In L1, the main GO enrichment terms included extracellular region (cellular component), oxidation − reduction process (biological process), and structural molecule activity (molecular function) (Fig. 5D). The KEGG enrichment analysis results showed that in L10, L4, and L1, pathways such as Regulation of lipolysis in adipocyte, Oxidative phosphorylation, and Fat digestion and absorption were significantly enriched (Fig. 6A-C). Additionally, genes related to Regulation of lipolysis in adipocyte, Oxidative phosphorylation, Fat digestion and absorption, and Fatty acid biosynthesis were significantly downregulated (Fig. S1). These results suggest that *P. pseudoannulata* may cope with hypoxic stress by regulating its energy metabolism.

### Association analysis of the metabolome and book lung transcriptome in *P. pseudoannulata* under hypoxic stress

**Fig. 7:**
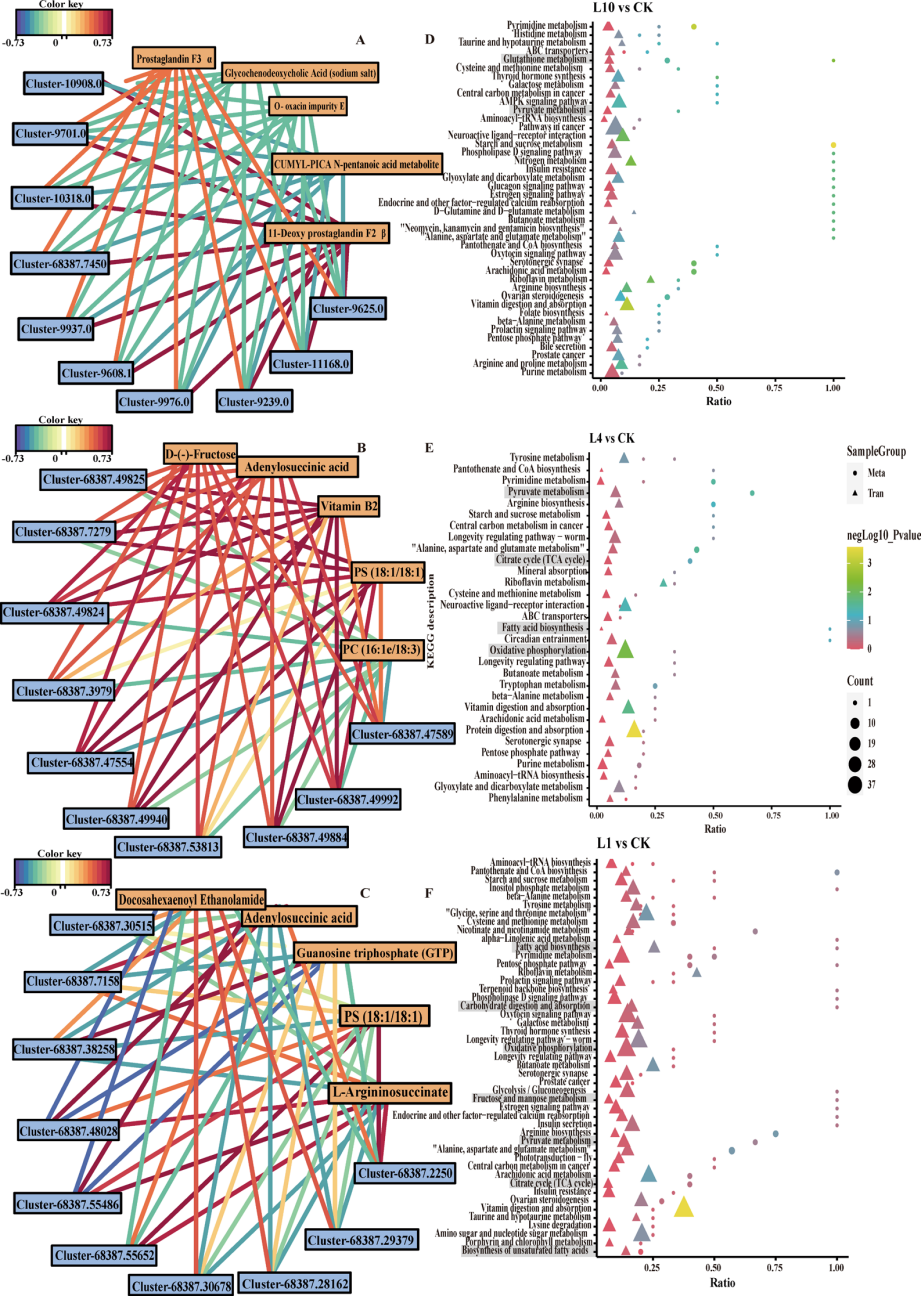
Transcriptome and metabolome correlation analysis. (**A**-**C**) CK vs. L10, CK vs. L4, CK vs. L1 correlation network diagrams. (**D**-**F**) CK vs. L10, CK vs. L4, CK vs. L1 coenrichment of KEGG pathways

We conducted correlation analysis between metabolites and transcripts to explore their relationship. The top 10 DEGs and the top 5 DEMs were selected for constructing network diagrams for visualization and analysis (Fig. 7A-C). Based on KO descriptions, our findings revealed that in L1, adenylosuccinic acid, GTP, and L-argininosuccinate were linked with chitinase, serine protease 27, transcription initiation factor TFIID subunit 15, and chromobox protein 3. This suggests that low-oxygen stress may impact the synthesis of energetic substances by regulating the biosynthesis of certain proteases, thereby influencing spider respiration.

In addition, we conducted KEGG enrichment analysis on metabolites and transcripts together (Fig. 7D-F). The results showed that, in L10, pathways significantly enriched included Glutathione metabolism and Pyruvate metabolism. In L4, pathways significantly enriched included Pyruvate metabolism, Citrate cycle (TCA cycle), Fatty acid biosynthesis, and Oxidative phosphorylation. In L1, pathways significantly enriched included Fatty acid biosynthesis, Carbohydrate digestion and absorption, Oxidative phosphorylation, Fructose and mannose metabolism, Pyruvate metabolism, Citrate cycle (TCA cycle), and Biosynthesis of unsaturated fatty acids. These pathways are mostly associated with energy metabolism and antioxidant functions, suggesting that metabolites and transcripts may collectively regulate antioxidant capacity and energy metabolism in response to hypoxic stress.

## Discussion

In the past decade, significant progress has been made in understanding the molecular mechanisms of the oxygen-sensing pathway. However, little is known about the molecular mechanisms underlying the response to hypoxia in invertebrates [25]. Arthropods can adapt to changes in environmental O_2_ levels by enhancing their antioxidant defenses [26–28]. However, hypoxia not only elevates reactive oxygen species (ROS) levels but also inhibits mitochondrial metabolism [29, 30]. Oxygen is a crucial factor driving cellular metabolism within mitochondria to maintain cellular energy homeostasis. It serves as the ultimate electron acceptor in oxidative phosphorylation, essential for energy production [31, 32]. Energy is vital for aerobic organisms to cope with hypoxic stress, including substrate metabolism, energy production, and mitochondrial biogenesis [27, 33, 34]. For instance, hypoxic stress weakens ATP turnover and may lead to suppressed glycolysis, downregulation of oxidative phosphorylation, and reduced activity of respiratory chain complexes [35–37]. Consequently, these effects have adverse impacts on animal survival and development.

In addition, insects can adopt various response strategies such as tracheal dilation, the HIF transcriptional regulatory system, and modulation of energy pathways to cope with hypoxic stress [38, 39]. In our study, we found that the abundance of various energy-related substances such as ATP, FAD, D-Glucose 6-phosphate, etc., significantly decreased, and numerous energy-related pathways were enriched. This is consistent with previous studies indicating that hypoxic stress can lead to disrupted energy metabolism in organisms [13, 40]. It has also been reported that hypoxic stress can lead to the accumulation of Reactive Oxygen Species (ROS) and oxidative damage to organisms, with Glutathione playing a crucial role in protecting cells from oxidative damage and serving as an important cofactor to promote the metabolism of energy substances [41, 42]. Similarly, in our study, we found a significant increase in the abundance of L-Glutathione (reduced), indicating that cells may activate their antioxidant defense systems to cope with oxidative stress under hypoxic conditions [43–45]. Additionally, Vitamin B2 is a precursor substance for the synthesis of FAD, and its cofactor derivatives (FAD, FMN) act as electron acceptors in the oxidation metabolism of carbohydrates, amino acids, and fatty acids, and in the reduced state, can provide electrons to complex II of the electron transport chain, implying the importance of Vitamin B2 for aerobic cells to generate energy through oxidative phosphorylation [46, 47]. Pantetheine is a functional subunit of Coenzyme A (CoA), and CoA serves as an important cofactor in numerous enzyme reactions involving energy production, lipid metabolism, and essential molecule synthesis [48, 49]. In our study, metabolomics detected a significant increase in the abundance of L-Malate, D-Fructose 6-phosphate, Vitamin B2, and Pantetheine. One possible explanation is that the metabolic processes of organisms are highly complex, and the levels of metabolites are influenced by multiple factors, including gene expression, protein activity, and cellular signal transduction, which may reflect complex metabolic interactions and may also be due to technical biases or sample processing reasons in metabolomics. KEGG enrichment results showed that pathways such as Oxidative phosphorylation, Fatty acid biosynthesis, Carbohydrate digestion and absorption were significantly enriched. This indicates that hypoxic stress can further affect the metabolism of *P. pseudoannulata* by influencing processes such as Carbohydrate and lipid metabolism [50]. Transcriptomic results also showed that energy metabolism-related pathways such as Regulation of lipolysis in adipocytes, Protein digestion and absorption, Oxidative phosphorylation were enriched, further demonstrating the importance of energy metabolism for *P. pseudoannulata* in coping with hypoxic stress.

Under hypoxic conditions, organisms struggle to obtain sufficient energy through aerobic metabolic pathways [51]. The first challenge for survival in low-oxygen environments is the balance between energy supply and demand. Many pathways related to energy and redox reactions were jointly enriched in the association analysis of the metabolome and transcriptome, indicating that *P. pseudoannulata* can respond to hypoxic stress by regulating its antioxidant capacity and energy metabolism.

## Conclusions

Overall, metabolic and transcriptomic analyses revealed that under hypoxic stress, *P. pseudoannulata* adapts by modulating energy substances, carbohydrates, cofactors, and antioxidants such as ATP, D-Glucose 6-phosphate, Flavin adenine dinucleotide (FAD), and L-Glutathione (reduced). Additionally, metabolic pathways including the Citrate cycle (TCA cycle), Glutathione metabolism, and Oxidative phosphorylation are regulated to cope with hypoxia. Transcriptomic analysis further confirmed these findings. In the transcriptome, pathways such as Regulation of lipolysis in adipocytes, Oxidative phosphorylation, Fat digestion and absorption, and Protein digestion and absorption were significantly enriched. Integrated analysis also highlighted that numerous pathways related to energy metabolism and antioxidant processes, like Glutathione metabolism, Pyruvate metabolism, and Oxidative phosphorylation, are significantly enriched, indicating that *P. pseudoannulata* primarily responds to hypoxic stress through energy metabolism and antioxidant pathways.

## Materials and methods

### Acquisition and handling of animal materials

In this study, a total of 192 adult female *P. pseudoannulata* spiders were collected from the experimental field of Hunan Agricultural University (113.08° E, 28.18° N). Each spider was individually housed in a glass test tube (1.5 × 10 cm, diameter × height) with a damp cotton ball at the bottom to provide water. Spiders were fed with 3–5 fruit flies daily and kept in an artificial climate chamber (26 °C, 70% RH, and L: D 10:14 light cycle). Subsequently, in the experimental phase, oxygen levels were lowered to approximately 1%, 4%, and 10% by injecting nitrogen gas (N_2_) into anaerobic incubators. In contrast, the CK group spiders were kept under normal air conditions in the climate chamber. Groups labeled L1, L4, and L10 were exposed to approximately 1%, 4%, and 10% oxygen concentrations for 30 min, respectively. Afterwards, spiders from these three groups and the CK group were dissected under a stereo microscope to obtain their book lungs for transcriptome analysis (with three replicates per group, each consisting of 10 book lungs). Similarly, intact spiders from each group were sampled for metabolomics analysis (with six replicates per group, each consisting of 3 spiders).

### Detection of spider metabolites by LC‒MS/MS

Spiders were ground in liquid nitrogen, and 100 mg of ground tissue samples were then placed in EP tubes. After adding 500 µl of 80% methanol aqueous solution, the samples were vortexed and mixed thoroughly, followed by centrifugation at 15,000 g and 4 °C for 20 min. The supernatant was collected and diluted with mass spectrometry-grade water to achieve a methanol content of 53%. After centrifugation, the supernatant was collected, and equal-volume samples were mixed from each experimental sample as QC samples. Additionally, 53% methanol aqueous solution was used as blank samples. Compounds were eluted using a linear gradient for 17 min, with methanol as the mobile phase in both positive and negative ion modes, at a flow rate of 0.2 mL/min. The samples were analyzed using a Hypesil Gold column (100 × 2.1 mm, 1.9 μm), coupled with a Vanquish UHPLC system (Thermo Fisher, Germany) and a Q Exactive™ HF mass spectrometer (Thermo Fisher, Germany). Compounds collected from both positive and negative ion modes were subjected to combined analysis. Differential metabolites were selected based on VIP > 1, p-value < 0.05, and fold change > 1.2 or fold change < 0.833 criteria, and their functions were investigated using the KEGG database.

Transcriptome sequencing of spider book lungs.

We extracted RNA from spider book lung tissues using the RNA extraction kit (Ambion, Austin, TX, USA) following the manufacturer’s instructions. Then, we assessed the integrity and quantity of RNA from the book lungs using the Agilent 2100 bioanalyzer. High-quality RNA samples were prepared for sequencing library construction using the NEBNext^®^ Ultra™ RNA Library Prep Kit, and were sequenced on an Illumina HiSeq 2500 platform to generate paired-end reads. Raw reads were filtered based on quality criteria including Q30, Q20, GC content, and sequence repetition levels. Subsequently, Trinity software (version 2.4.0) was employed to assemble transcripts and unigenes [52]. Differential gene expression analysis was conducted using the DESeq2 R package, with Benjamini-Hochberg (BH) correction applied to obtain false discovery rate (FDR) values. Differential genes were filtered using thresholds of |log2(FoldChange)| > 1 and FDR < 0.05. GOseq (version 1.10.0) and KOBAS (version2.0.12) software were utilized for Gene Ontology (GO) and Kyoto Encyclopedia of Genes and Genomes (KEGG) pathway enrichment analyses of differentially expressed genes.

### Statistical analysis

Transcriptomic sequence analysis was conducted by NCBI (https://www.ncbi.nlm.nih.gov/). The R package pheatmap (https://www.r-project.org/) was used to determine the expression level of identified genes. Differential metabolites were subjected to t-tests using GraphPad Prism 8.0 software, with results presented as mean ± SEM. Pearson correlation coefficient was employed to measure the relationship between metabolites and transcripts, and the results were visualized using the “ggcorrplot” package in R. A significance level of *P* < 0.05 was applied to indicate statistical significance. Adobe Illustrator CC was utilized for graphical processing.

*P. pseudoannulata: Pardosa pseudoannulata*.

L10: 10% low oxygen concentration.

L4: 4% low oxygen concentration.

L1: 1% low oxygen concentration.

### Electronic supplementary material

Below is the link to the electronic supplementary material.

Supplementary Material 1

## Data Availability

No datasets were generated or analysed during the current study.
